# Calcium-Related Gene Signatures May Predict Prognosis and Level of Immunosuppression in Gliomas

**DOI:** 10.3389/fonc.2022.708272

**Published:** 2022-05-13

**Authors:** Peidong Liu, Yu Li, Yiming Zhang, John Choi, Jinhao Zhang, Guanjie Shang, Bailiang Li, Ya-Jui Lin, Laura Saleh, Liang Zhang, Li Yi, Shengping Yu, Michael Lim, Xuejun Yang

**Affiliations:** ^1^ Department of Neurosurgery, Tianjin Medical University General Hospital, Tianjin, China; ^2^ Department of Neurosurgery, Stanford University School of Medicine, Palo Alto, CA, United States; ^3^ Laboratory of Neuro-Oncology, Tianjin Neurological Institute, Tianjin, China; ^4^ Department of Radiation Oncology, Stanford University School of Medicine, Palo Alto, CA, United States; ^5^ Department of Neurosurgery, Chang Gung Memorial Hospital, Linkou, Taiwan; ^6^ Department of Neurosurgery, Beijing Tsinghua Changgung Hospital, Tsinghua University, Beijing, China

**Keywords:** calcium-related genes, risk signature, glioma, immunosuppressive microenvironment, unsupervised/supervised learning

## Abstract

Gliomas are the most common primary brain cancer. While it has been known that calcium-related genes correlate with gliomagenesis, the relationship between calcium-related genes and glioma prognosis remains unclear. We assessed TCGA datasets of mRNA expressions with differentially expressed genes (DEGs) and enrichment analysis to specifically screen for genes that regulate or are affected by calcium levels. We then correlated the identified calcium-related genes with unsupervised/supervised learning to classify glioma patients into 2 risk groups. We also correlated our identified genes with immune signatures. As a result, we discovered 460 calcium genes and 35 calcium key genes that were associated with OS. There were 13 DEGs between Clusters 1 and 2 with different OS. At the same time, 10 calcium hub genes (CHGs) signature model were constructed using supervised learning, and the prognostic risk scores of the 3 cohorts of samples were calculated. The risk score was confirmed as an independent predictor of prognosis. Immune enrichment analysis revealed an immunosuppressive tumor microenvironment with upregulation of checkpoint markers in the high-risk group. Finally, a nomogram was generated with risk scores and other clinical prognostic independent indicators to quantify prognosis. Our findings suggest that calcium-related gene expression patterns could be applicable to predict prognosis and predict levels of immunosuppression.

## Introduction

Gliomas are the most common primary cancer of the central nervous system (CNS). Based on the 2016 diagnostic criteria of the World Health Organization (WHO), glioma is classified into 4 grades primarily through molecular pathology. Grade I has the lowest proliferative ability with the best prognosis, while grades II–IV demonstrate increasing malignancy with worsening prognosis. Therefore, diffuse glioma samples, including grades II–IV, were employed in the research. Grade IV tumors comprise glioblastoma (GBM), which is a highly aggressive brain tumor that comprises 47.1% (1) of the CNS malignant tumors. GBMs are highly aggressive and are resistant to traditional interventions including surgical resection followed by chemotherapy and radiotherapy. The 5-year survival rate is 5.1% (1), and the 10-year survival rate is 0.17% (2).

Calcium ions play a vital role not only in normal physiological processes but also in many cancers including gliomas. Calcium plays an important role in intracellular cell signaling (3–5), ion channel potentials (6, 7), cell death and proliferation (5, 8), calcium-binding proteins (3, 8, 9), cellular homeostasis (10), autophagy (11, 12), and synaptic plasticity/junctions (10, 13, 14). These calcium-mediated processes also play an important role in the tumorigenesis of cancers such as GBM. Néant’s study (15) reported that calcium ion signaling would be an important regulator of tumorigenesis in GBM. The transition from glioma proliferation to quiescence would involve the modification of the kinetics of calcium ion influx due to an increased capacity of the mitochondria of quiescent GSLC (glioblastoma stem-like cells) to capture calcium ions, which would benefit to new therapeutic strategies. Therefore, we hypothesized that calcium-related genes could be closely associated with the progression and prognosis of gliomas. A comprehensive study associating calcium-related genes in glioma patients with OS has yet to be explored.

Therefore, this study screened calcium genes in TCGA cohort to assess for associations with overall survival (OS) from the most prevalent calcium-related pathways. A multigene-independent prognostic indicator was generated following unsupervised/supervised learning in both the training (TCGA) cohort and 2 independent external validation (CGGA, Rembrandt) cohorts. We also followed this up with immune-related enrichment analysis. We also attempted to create a prognostic predictive tool to predict survival and propensity for immunosuppression.

## Materials and Methods

### Datasets and Samples

TCGA mRNA HTseq Counts and FPKM datasets, including clinical information, were downloaded from TCGA website (https://portal.gdc.cancer.gov) by R project (v 4.0.2) and R package “TCGAbiolinks” (v2.16.4). Three hundred and twenty-five-CGGA-glioma mRNA expression datasets and corresponding clinical information were downloaded from the CGGA website (https://www.cgga.org.cn). The Rembrandt dataset involving mRNA microarray and clinical data was downloaded from Betastasis (http://www.betastasis.com/). Glioma and control tissues were those of clinical excision brain obtained from the Tianjin Medical University General Hospital. These tissue samples underwent immunohistochemistry (IHC) to study the immune microenvironment of glioma.

### Differential Expression Gene Analysis

TCGA HTSeq-Counts cohort was divided into lower-grade glioma (LGG), GBM, and (normal control tissue) NT groups according to clinical information; differential expression genes (DEGs) were compared in LGG-NT, GBM-NT, and GBM-LGG groups by “DESeq2”. p.adjust < 0.05 and |log2(FoldChange)| > 2 were set as the cutoff thresholds to screen DEGs. We screened for 1,171 DEGs (TCGA-DEGs) in the GBM-LGG (GL) group, 4,077 in the GBM-NT (GN) group, and 1,858 in the LGG-NT (LN) group. We finally selected TCGA-DEGs in the FPKM dataset for future analysis.

### Enrichment Analysis

All gene enrichment analyses were done with R package “clusterProfiler” (v 3.16.0), including Gene ontology (GO), Kyoto Encyclopedia of Genes and Genomes (KEGG), and gene set enrichment analysis (GSEA) with the GO/KEGG database. Significantly changed calcium-related biological processes (BP), molecule function (MF), and pathways were screened. We then obtained union sets of GO terms in calcium-related BPs/MFs, and genes involved in those terms were obtained. Protein–protein interaction network (PPI) analysis was performed with selected genes in our study *via* the STRING database (https://string-db.org).

### Unsupervised Learning

We performed unsupervised learning to cluster glioma samples into several clusters with consensus clustering by R package “ConsensusClusterPlus”(v 1.52.0). We set 10,000 iterations, a resample rate of 85%, clustering algorithm of “k-means,” and distance function of “euclidean” to conduct consensus clustering. The best clustering number, *k* value, was verified by both consensus clustering result and validation testing by R package “fpc”(v 2.2-8). When the cutoff threshold was set as 0.8 with method of “k-means,” the best *k* value was 2, which followed the consensus clustering result. Next, we performed principal component analysis (PCA) and t-distributed stochastic neighbor embedding (tSNE) with R package “PCAtools” (v 2.0.0) and “Rtsne” (v 0.15), to verify consensus and to further study the calcium key genes (CKGs) expression patterns in different glioma Clusters 1 and 2.

### Supervised Learning

To estimate and predict OS with crucial genes and prognostic indicators, the cox proportional hazard regression model was used. Univariate Cox (Uni-Cox) regression analyses were operated with R package “survival”(v 3.2-3) to screen calcium-related genes that were statistically significant (p < 0.001) in their association with prognosis. Finally, thirty-five genes were acquired, as calcium key genes (CKGs), for further analysis. The Lasso regression algorithm was conducted with R package “glmnet”(v 4.0-2) to develop a survival prediction model with a potential risk signature. The best penalty parameter λ was selected and associated with the smallest cross-validation error within the training set. We then obtained 10 genes as calcium hub genes (CHGs). The prediction algorithm in R package “stats” was used to calculate risk scores for each sample among the training cohort (TCGA) and external independent validation cohorts (CGGA and Rembrandt). We divided glioma samples into 2 groups based on the median of risk scores produced by the Lasso regression algorithm and found 9 DEGs in calcium hub genes (CHGs) between those 2 groups. Finally, lasso risk scores were combined with univariate Cox (Uni-Cox) regression to screen for clinical features. According to the Uni-Cox results, the Multivariate-Cox (Multi-Cox)/logistical regression model was constructed for prognostic estimation *via* R package “rms”(v 6.0-1). The calibration curve was created with R package “Hmisc”(v 4.4-0).

### Statistics

The DESeq2 algorithm was used to compare calcium gene expression levels in different classifications. We assessed the overall survival (OS) of each cohort mentioned above and removed samples without OS or survival status (SS). Survival analysis was conducted by the Kaplan–Meier method with a 2-tailed log-rank test to compare OS in samples with different groups. The median gene expression value was conducted as a cutoff threshold for survival analysis. The Mantel–Cox test was performed for survival-related analysis, with p < 0.05 considered statistically significant. Survival analyses were done on R project with R package “survival” and “rms.” The predicted efficiency of the risk signature for 1/3/5-year survival was tested by the receiver operating characteristic (ROC) curve with R package “survivalROC” (v 1.0.3). Pearson correlation coefficients for CKGs were calculated *via* R package “stats” (v 4.0.2). Statistics hypothesis testing was validated in SPSS 21.0 (IBM Corporation, 1 New Orchard Road Armonk, NY 10504, USA) and was accordant with the R project.

## Results

### Calcium Key Genes Were Screened *via* Multiple-Enrichment Analysis and Prognosis-Related Analysis

**Figure 1:**
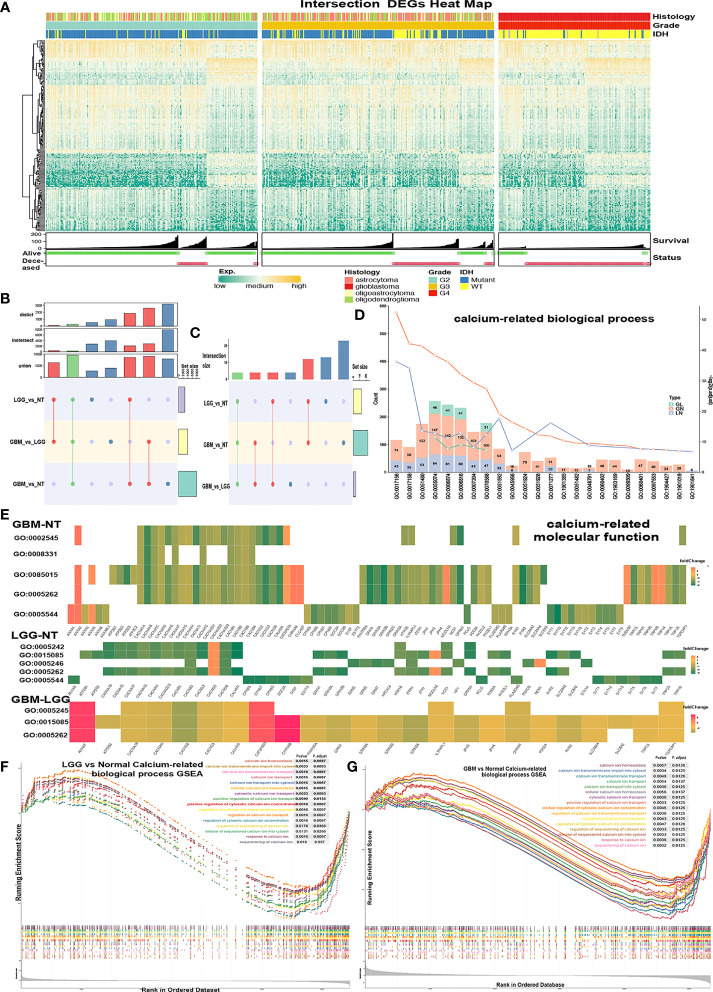
Screening calcium-related enrichment terms for obtaining calcium genes. **(A, B)** Intersect DGEs (TCGA-DEGs, p < 0.05, log2(FoldChange) > 2) of GBM-NT, LGG-NT, and GBM-LGG. **(C–E)** Enrichment analysis of the DEGs in significantly (p < 0.05) enriched calcium-related GO biological processes terms. **(C)** The count of enriched terms among the GBM-NT/GBM-LGG/LGG-NT groups. **(D)** The gene count of each calcium-related term was marked on each color bar (green = GBM-LGG, red = GBM-NT, purple = LGG-NT). **(E)** Enrichment analysis heatmap of the TCGA-DEGs in significantly (p < 0.05) enriched calcium-related GO molecular function terms. **(F, G)** Gene-set enrichment plots show enriched calcium-related terms (p < 0.05) in the GBM-NT group **(G)** and LGG-NT **(F)**.

Lower-grade glioma (LGG) samples, GBM samples, and normal control tissue samples (NT) were examined for analysis. Three groups were matched against each other: GBM versus LGG (GL), GBM versus NT (GN), and LGG versus NT (LN). Gene expression count values were compared between each group, and genes matching criteria of p.adjust < 0.05 and |log2Foldchange| > 2 were regarded as differentially expressed genes (DEGs). In total, 7,942 genes in the group of GN, 3,970 genes in GL, and 2,765 in LN (**Supplementary Figure 1A**) were screened. 289 intersect DEGs were screened among 3 groups mentioned above (**Figure 1B** and **Supplementary Figure 1B**). The intersection DEG (TCGA-DEG) heatmap (**Figure 1A**) shows a differential gene expression pattern according to survival status (SS), OS, and IDH status.

Gene ontology (GO) and Kyoto Encyclopedia of Genes and Genomes (KEGG) enrichment analyses were conducted separately with TCGA-DEGs in the GN, GL, and LN groups. Significantly enriched biological process (BP), molecular function (MF), and pathways were regarded as p.adjust < 0.05 and were recorded for further analysis. Terms of BP and MF related to calcium were selected (**Figures 1C, E**). There were 23 BP/5 MF terms of GN, 13 BP/5 MF terms of LN, and 4 BP/3 MF terms of GL. There are 4 intersected BP terms (**Figure 1D**), GO0055074 (calcium ion homeostasis), GO0006874 (cellular calcium ion homeostasis), GO0006816 (calcium ion transports), and GO0070588 (calcium ion transmembrane transport), and 3 intersected MF terms: GO0015085 (calcium ion transmembrane transporter activity), GO0005262 (calcium channel activity), and GO0005245 (voltage-gated calcium channel activity). The gene expression patterns of calcium MF/BP terms in 3 groups were shown in the enrichment heatmap (**Figure 1E** and **Supplementary Figure 1E**). Hsa04020 (calcium signaling pathway) was also enriched in KEGG (**Supplementary Figure 1C**). Of note, hsa04961 (endocrine and other factor-regulated calcium reabsorption pathway) was enriched only in the LN group (**Supplementary Figure 1D**).

Gene-set enrichment analysis (GSEA) was performed with all genes, and terms with p.adjust < 0.05 were regarded as significantly enriched. We obtained 18 BP/2 MF terms in GN (**Figure 1G** and **Supplementary Figure 1F-I**), 17 BP/2 MF terms in LN (**Figure 1F** and **Supplementary Figure 1F-II**).

**Figure 2:**
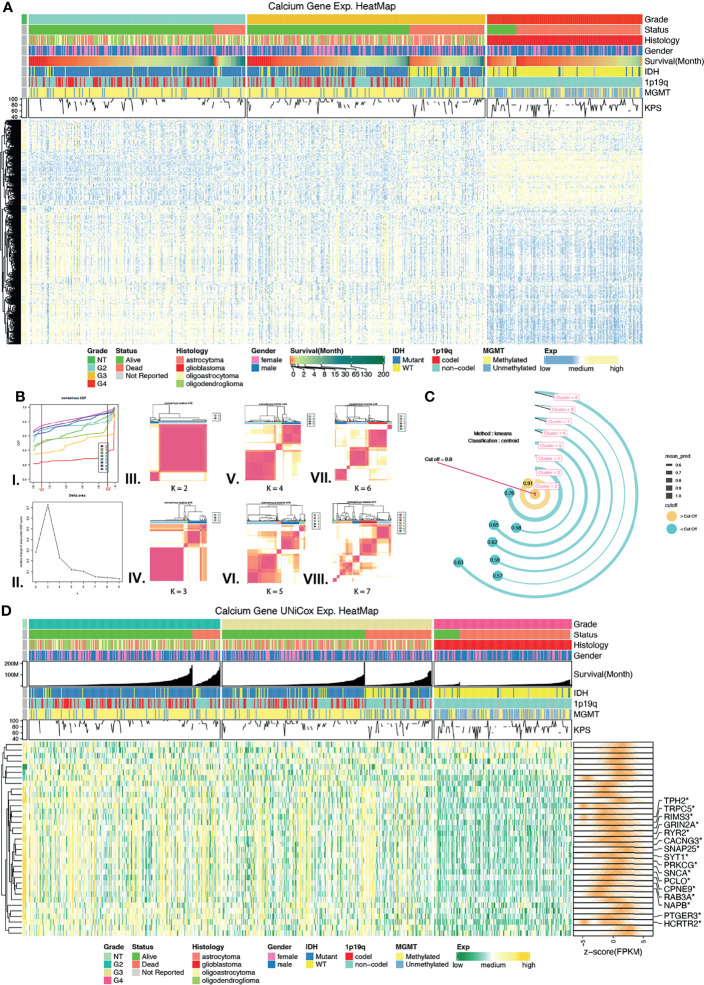
Screening calcium key genes (CKGs) followed by unsupervised learning. **(A)** Calcium Genes expression and clinical features heatmap among normal control tissues, grade 2, grade 3, and grade 4 glioma. **(B)** Unsupervised learning of consensus clustering with CKGs, (I.) Consensus CDF line chart, (II.) Consensus delta area line chart, (III–VIII.) Consensus matrixes when k value varies from 2 to 7. **(C)** External validation method of consensus clustering, bar in yellow color is prediction value greater than threshold value (0.8). **(D)** Heatmap for CKGs extremely associated with overall survival (p < 0.001, Univariate Cox regression) and marked in DEGs. *p < 0.05.

To obtain the calcium genes, we selected the calcium-related union terms of GO results and GSEA results (**Supplementary Table 2**). There were 312 calcium-related genes in union genes of GO terms and 330 in GSEA terms. Then, union genes between these 2 sets were screened. As a result, we obtained 460 genes as calcium genes (**Figure 2A**). We found that these 460 calcium genes were also collected by the Reactome database with the calcium-related pathway (https://reactome.org/).

Univariate Cox (Uni-Cox) regression analysis was performed to screen calcium genes that were significantly related to OS. Thirty-five genes (p < 0.001) were selected as calcium key genes (CKGs) (**Figure 2D** and **Supplementary Table 1**), and TCGA-DEGs in 3 groups were marked *via* a heatmap.

### Clustering of CKGs Identified Cluster 1/2 Related With Clinical Prognosis

Based on the consensus expression pattens of CKGs, glioma samples were clustered into several clusters (**Figure 2B** and **Supplementary Figures 2A–F**). In the CDF curve, which was designed to measure the stability of consensus matrices, the lower left portion represents samples that rarely clustered together while the upper right portion represents samples that always clustered together. The middle portion represents those with ambiguous assignments in different clustering runs. The lowest proportion of ambiguous clustering (PAC) was “k = 6,” which was followed by “k = 2.” However, from the consensus matrix we found when selecting “k = 6,” there was a lower correlation within the cluster than the matrix with “k = 2,” as well as some clusters with “very small samples.” Therefore, we decided to use another method to determine what would be the best “k” value. “Prediction Strength” created by Robert Tibshirani et al. was developed to solve this situation. We used the same central algorithm of “k-means” as consensus clustering and the classification method of “centroid” to calculate the mean prediction value of “k” which varied from 2 to 9. When the cutoff was set to the default value (0.8), the largest number of clusters better than the cutoff was two. As such, we determined the optimal matrix setting of k = 2 (**Figure 2C**) and clustered the TCGA glioma samples into two clusters: Cluster 1 and Cluster 2 cohorts.

**Figure 3:**
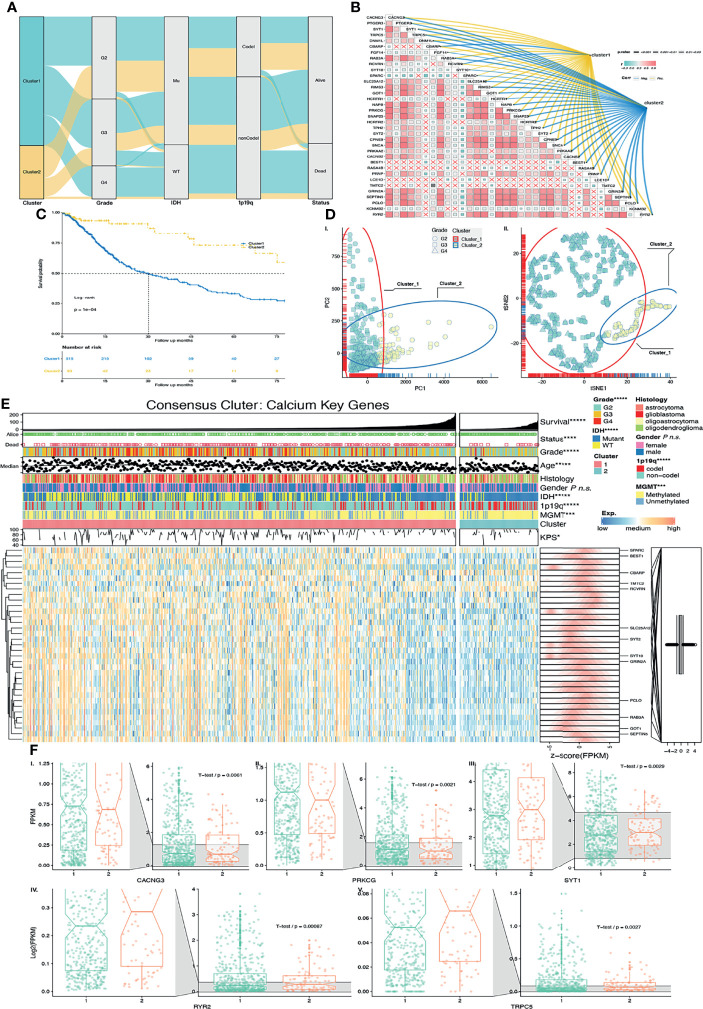
Validation of unsupervised learning. **(A)** Alluvial diagram of CKG clustering distribution in groups with different clusters, glioma grade, IDH status, 1p19q codeletion status, and survival outcomes. **(B)** Correlation among CKGs was exhibited in a heatmap. Correlation between CKGs and clusters was exhibited on colored (positive correlation = yellow, negative correlation = blue) links with p values as size of line. **(C)** Kaplan–Meier curves for Cluster 1/2 in TCGA cohort. Log-rank test, p = 0.0001. **(D)** External unsupervised learning method of (I.) PCA and (II). tSNE-validated consensus clustering. Clusters 1/2 and glioma grades were mapping in different colors and shapes of the points. **(E)** Heatmap of CKGs with clinical features and clusters. TCGA-DEGs were marked. **(F)** CKG expression values with significant expression between Cluster 1/2 (I–V.). *p < 0.05; ***p < 0.001; ****p < 0.0001; *****p < 0.00001; ns p > 0.05.

To evaluate whether the results of unsupervised learning are clinically significant, we performed chi-square tests to compare the distribution of gender, grade, age, IDH status, 1p19q co-deletion status, MGMT promoter methylated, and the Karnofsky Performance Scale (KPS) between clusters 1 and 2 (**Figure 3E**). Cluster 2 demonstrated findings consistent with lower grade (p < 0.00001), IDH1 mutant (p < 0.00001), 1p19q co-deletion status (p < 0.00001), MGMT promoter methylated (p < 0.001), age < 65 years old (p < 0.05), and higher KPS scores (p < 0.05). Notably, Cluster 2 also had better overall survival (OS) than Cluster 1 (**Figure 3C**). In short, samples associated with better prognosis were found in Cluster 2 based on unsupervised learning. Evidence from Sankey analysis (**Figure 3A**) also confirmed that Cluster 2 includes more LGG samples (especially, in grade 2) and more IDH1 mutant samples, which are both better prognostic indictors.

We also performed principal component analysis (PCA) and t-distributed stochastic neighbor embedding (tSNE) to compare the transcriptional profile in CKGs. Similarly, in PCA (**Figure 3D-I**) and tSNE (**Figure 3D-II**), glioma samples were gathered into 2 subgroups (especially in PC1 and tSNE1). The blue color ellipse and points in yellow-green color are the Cluster 2 samples that are mostly composed of LGGs (in shape of square and round).

The discoveries above strongly suggest that consensus clustering results are closely related with patient prognosis with glioma. To further study the expression pattern of calcium key genes, we did a correlation analysis and found more than half of those genes to have the trend of correlation (**Figure 3B**). Furthermore, we compared the CKG correlation with Cluster 1/2. The links in blue color are negative with Cluster 1/2, and links in yellow color are positive with Cluster 1/2 (**Figure 3B**). We found those genes to have a similar expression feature with Cluster 1/2.

Next, we selected CKGs that belong to the union set of DGEs and compared their expression within Cluster 1/2. Six genes were significantly changed (p < 0.05), between Clusters 1 and 2 (**Figure 3F**). The expression values of CACNG3, and PRKCG in Cluster 1 are higher than Cluster 2 with p < 0.05. However, the expression values of RYR2, SYT1, and TRPC5 in Cluster 1 are lower than those in Cluster 2. As we described above, patients in Cluster 1 have poorer survival than those in Cluster 2. Based on that, we hypothesized that the genes of CACNG3 and PRKCG are probably promoting gliomagenesis while genes RYR2, SYT1, and TRPC5 are probably suppressing gliomagenesis.

### Constructing Prognostic Risk Signature Followed by Screening Calcium Hub Genes

**Figure 4:**
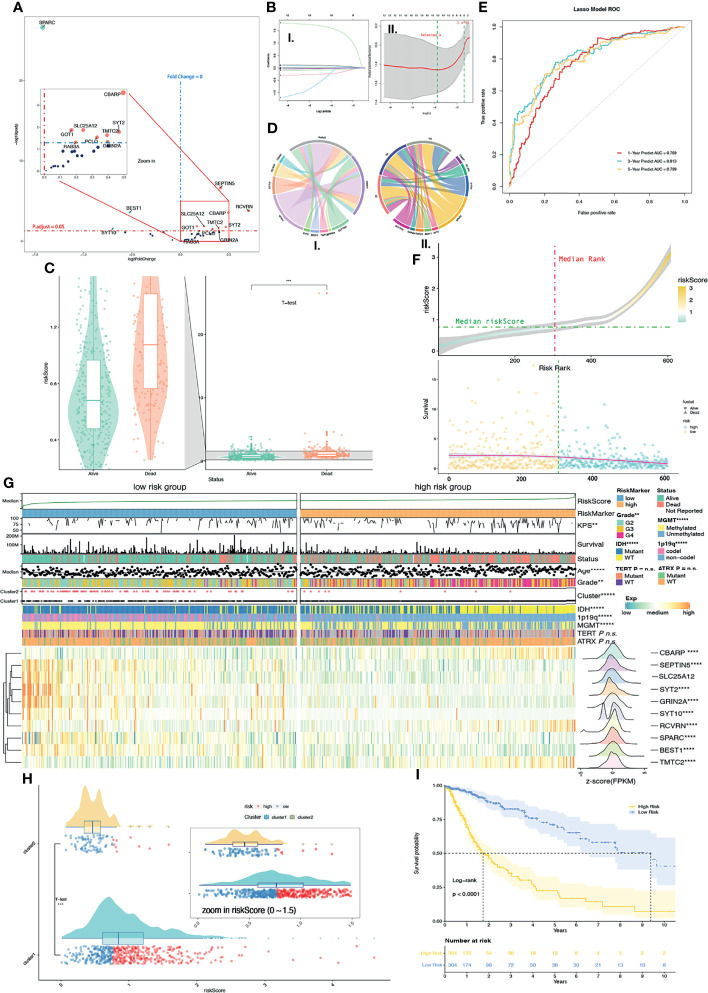
Supervised learning of DEGs in CKGs between Cluster 1/2. **(A)** 13 DEGs were found in CKGs between Cluster 1/2 (p.adjust < 0.05). Upregulated genes were marked in red color, and downregulated genes were marked in green color. **(B)** Supervised learning with Lasso–Cox arithmetic (I.) and (II.) λ was selected when partial likelihood deviance came to smallest (II. left green line). **(C)** Diagram of survival outcomes with risk score (p < 0.001). **(D)** Mean expression values of DEGs in calcium hub genes (CHGs) between high/low-risk groups (I.) and among glioma grades (II.). **(E)** ROC curve showed the predictive efficiency of the 1/3/5-year survival rate on risk score (1-year AUC = 0.769, 2-year AUC = 0.813, 3-year AUC = 0.799). **(F)** Distribution of risk score with risk rank (upper plot) and overall survival (lower plot). **(G)** The heatmap shows the expression levels of the CHGs in low/high-risk groups and the significantly (p < 0.05) changed gene was marked. The distribution of clinical features was compared between the low-risk and high-risk groups. **(H)** Risk score levels with Cluster 1/2. **(I)** Kaplan–Meier overall survival curves for high/low-risk groups. Log-rank test, p < 0.0001. n.s. p ≥ 0.05, **p < 0.01, ***p < 0.001, ****p < 0.0001, and *****p < 0.00001.

We performed differential expression calcium key gene (DECKG) analysis between Clusters 1 and 2 and found 13 DECKGs by setting threshold p.adjust < 0.05 (**Figure 4A**). Then, we used those genes with TCGA cohort (n = 608) glioma patients’ OS and SS (as the training cohort) to build the prognosis risk signature model *via* the lasso regression algorithm. We regarded λ as the best when partial likelihood deviance is at the minimum value (**Figure 4B**). We then found 10 genes (marked as calcium hub genes, CHGs) that were screened by this algorithm with each gene’s coefficient, which could be further used to calculate the risk score for each sample. The mean expression values of CHGs, which demonstrate a differential expression between low-/high-risk groups with Cluster 1/2 grades, were displayed on circus plots (**Figure 4D I–II**).

The ROC curve shows that the risk score of each glioma sample can satisfactorily predict 1-year (AUC = 0.769), 3-year (AUC = 0.813), and 5-year (AUC = 0.799) survival rates (**Figure 4E**). Furthermore, we also compared SS based on risk score and significantly found that deceased patient samples had a much higher risk score than alive patient samples with the T-test (p < 0.0001) (**Figure 4C**). The median of TCGA glioma samples’ risk score was used as a cutoff to divide TCGA glioma samples into a low-risk group and high-risk group (**Figure 4F**). Meanwhile, we further compared the relationship with Cluster 1/2 and the risk score. Results showed that the Cluster 2 samples with good prognosis correlated with the low-risk group and more than half of the Cluster 1 samples were in the high-risk group by the T-test (p < 0.0001) (**Figure 4H**). The survival curve based on high-/low-risk groups also showed significantly (p < 0.0001) longer OS in the low-risk group than in the high-risk group (**Figure 4I**).

We then compared, by the chi-square test, the KPS, age, grade, Cluster 1/2, IDH status, 1p19q codeletion, MGMT promoter methylation status, TERT promoter status, and ATRX status between low-/high-risk groups, and results were shown on a heatmap (**Figure 4G**). Significant differences between the high-/low-risk groups are KPS (p < 0.01), age (p < 0.00001), grade (p < 0.01), Cluster 1/2 (p < 0.0001), IDH status (p < 0.00001), 1p19q codeletion (p < 0.00001), and MGMT promoter methylation status (p < 0.00001), but not TERT promoter status and ATRX status. A larger number of missing values (**Figure 4G**) result in no significant differences in TERT promoter status (missing values on the heatmap are shown in gray). DEG analysis of 10 CHGs was conducted between the high-/low-risk groups by their count values, and 9 genes with p.adjust < 0.05 were marked with asterisks on the heatmap (**Figure 4G**). Expression features were transformed into normal distribution and were also shown on row annotation in the heatmap. The gene expression distribution shows genes of CBARP (fold change, FC = 1.78), RCVRN (FC = 1.77), SLC25A12 (FC = 1.02, p.adjust = 0.58), and TMTC2 (FC = 2.00) which were upregulated in the high-risk group and SEPTIN5 (FC = 0.67, also shown as SEPT5 in other datasets), SYT2 (FC = 0.38), GRIN2A (FC = 0.52), SYT10 (FC = 0.26), BEST1 (FC = 0.66), and SPARC (FC = 0.73) in the low-risk group.

### Risk Score Efficiently Predicts Prognosis

**Figure 5:**
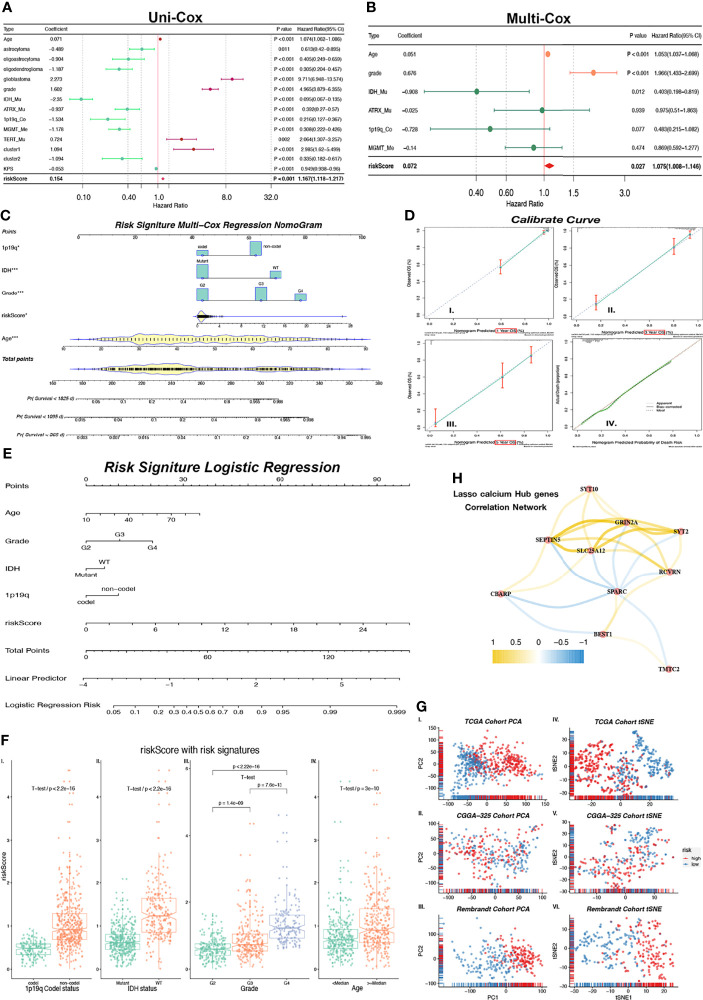
Nomogram constructed with independent predictor of risk core, 1p19q, IHD, Grade and age. **(A)** Forest plot shows indicators HR values calculated by Uni-Cox regression. HR < 1 is in green color and HR > 1 is in purple color. **(B)** Forest plot shows P and HR values calculated by Multi-Cox regression with clinical indicators (Age, Grade, IDH, ATRX, 1p19q, MGMT) and risk score. HR < 1 is in green color and HR > 1 is in red color. Indicators with P < 0.05 are considered as glioma independent predictor. **(C)** Multi-Cox regression Nomogram of 1/3/5-year predicted survival with independent predictor. **(D)** Calibrate curves of Multi-Cox regression Nomogram (I-III.) and logistic regression Nomogram (IV.). **(E)** Logistic regression Nomogram of predicted risk degree with independent predictor. **(H)** Correlation expression network with GHGs. Positive correlation is in yellow color, and negative correlation is in blue color. **(F)** Risk score levels with risk signatures (1p19q, IDH, Grade, Age). **(G)** Unsupervised clustering (PCA: I-III. and tSNE: IV–VI.) of train (TCGA) cohort and external independent validation (CGGA-325 and Rembrandt) cohort show satisfied classification of glioma patients according to low/high-risk groups. *p < 0.05; ***p < 0.001.

In order to confirm whether risk scores can better predict the prognosis of glioma patients, we first performed Uni-Cox regression with risk score, clinic-related features, and Cluster 1/2. The forest plot of Uni-Cox regression shows that age, GBM, grade (higher), TERT promoter status of mutant, Cluster 1, and risk score (higher) are harmful to prognosis, with hazard ratios (HR) > 1 (**Figure 5A**). Glioma pathologies of astrocytoma, oligoastrocytoma, and oligodendroglioma which are usually cosigned as LGGs are correlated with better prognosis than GBM. IDH and ATRX mutant status also demonstrated a positive correlation with OS with HR < 1. MGMT promoter methylation status and 1p19q co-deletion are molecular features of good prognosis in glioma, and patients with methylation on MGMT promoters usually have a better response to chemotherapy. Their HRs are both > 1. Cluster 2 shows better prognosis (HR < 1) while Cluster1 is associated with poorer prognosis with HR > 1. The KPS is used to classify the patients’ health status, and a higher score represents a better prognosis (16). In this study, we validated that the HR of KPS is less than 1 (**Figure 5A**). All variates that we used in Uni-Cox regression had a p value less than 0.05. Meanwhile, we selected risk scores and clinical–pathological variates that were related to prognosis with p < 0.001 for Multi-Cox regression. We observed that only age, grade, IDH status mutant, and risk score remain significantly associated with OS (**Figure 5B**). The correlation network between 10 calcium hub genes was computed by “Pearson” correlation, and SEPTIN5 (also SEPT5), SLC25A12, and GRIN2A were the node genes in this network (**Figure 5H**). Interestingly, the gene of SPARC had the most negative relationship with the other genes, which indicates that this gene has the opposite function as compared with the others. SPARC has a higher expression among lower grade (**Supplementary Figure 3A**), Cluster 2 (**Supplementary Figure 3B**), low-risk group (**Supplementary Figure 3C**), IHD status of mutant (**Supplementary Figure 3D**), GBM subtype of mesenchymal (**Supplementary Figure 3E**), and IDH status of mutant in LGG (**Supplementary Figure 3E**) groups.

We also calculated the risk score in both of the CGGA-325 and Rembrandt validation cohorts. The survival curves of these 2 cohorts show a significant difference (p < 0.0001) between high- and low-risk groups (**Supplementary Figures 4A, F**). Meanwhile, we observed that the ROC curves of risk score in both the CGGA-325 (1/3/5-year AUC = 0.629/0.698/0.745) and Rembrandt (1/3/5-year AUC = 0.634/0.705/0.681) cohorts had satisfactory outcomes for 1/3/5-year survival prediction (**Supplementary Figures 4B, G**). Robust evidence correlating risk score with prognosis was observed from Multi-Cox regressions (**Supplementary Figures 4D, I**) in CGGA-325 (p < 0.001) and Rembrandt (p < 0.01). Through these results, we confirmed that the risk score is an independent predictor of prognosis in patients with glioma.

### Prognostic Risk Predicted Nomogram Based on Risk Score Is a Promising Glioma Survival Predicted Tool

As observed above, we selected risk score and clinical features with p < 0.05 in Multi-Cox regression to build a prognostic predicted nomogram. Although the p value of the 1p19q codeletion status is 0.07 which is greater than 0.05, there is currently much evidence that demonstrates that the 1p19q codeletion status is closely associated with patient prognosis (17). Therefore, 1p19q is considered as one of risk signatures in our study. The KPS score is another prognosis-related indicator that is mostly used as an attempt to quantify patients’ overall morbidity from disease. Generally, a higher KPS score is associated with longer survival (16). This score is used in the clinical setting to help evaluate for candidacy for receiving chemotherapy and evaluating therapy response. However, KPS is not used as a prognostic predictor in clinical activity. The KPS score has complete criteria, but it is based on manual scoring. Therefore, KPS is inappropriate as a risk signature predictor in our study based on the objective variable analysis. Finally, age, grade, IDH status, and 1p19q codeletion status were selected to build a nomogram model. We use Multi-Cox and logistic regression to set up the model separately, and they were displayed in the nomogram (**Figures 5C, E**). The Multi-Cox regression nomogram can predict the 1/3/5-year survival, and the logistic regression nomogram can predict the prognostic risk value.

The multivariable regression calibration curves show outstanding precision of regression models both in 1/3/5-year survival (**Figure 5D I-III**) and prognosis risk (**Figure 5D-IV**). Similar results were found in the external independent validation cohorts of CGGA-325 and Rembrandt (**Supplementary Figure 4C–E, H–J**). We also observed that the risk score has a significant difference between 1p19q codeletion status (**Figure 5F-I**, p < 2.2 × 10^-16^), IDH status (**Figure 5F-II**, p < 2.2 × 10^-16^), grade (**Figure 5F-III**, all p < 1.4 × 10^-9^), and age (**Figure 5F-IV**, all p = 3 × 10^-10^). In conclusion, the risk score produced by calcium hub genes is closely related to clinical molecular pathological features, and the multivariate regression nomogram can be used as a tool to predict glioma prognosis. Results generated by PCA and tSNE provide another strong evidence that risk score can divide glioma patients into low-/high-risk groups among TCGA training cohort and validation cohorts of CGGA-325/Rembrandt (**Figure 5G I-VI**).

### Tumor-Immunosuppressive Microenvironment Enriched in High-Risk Glioma Patients

**Figure 6:**
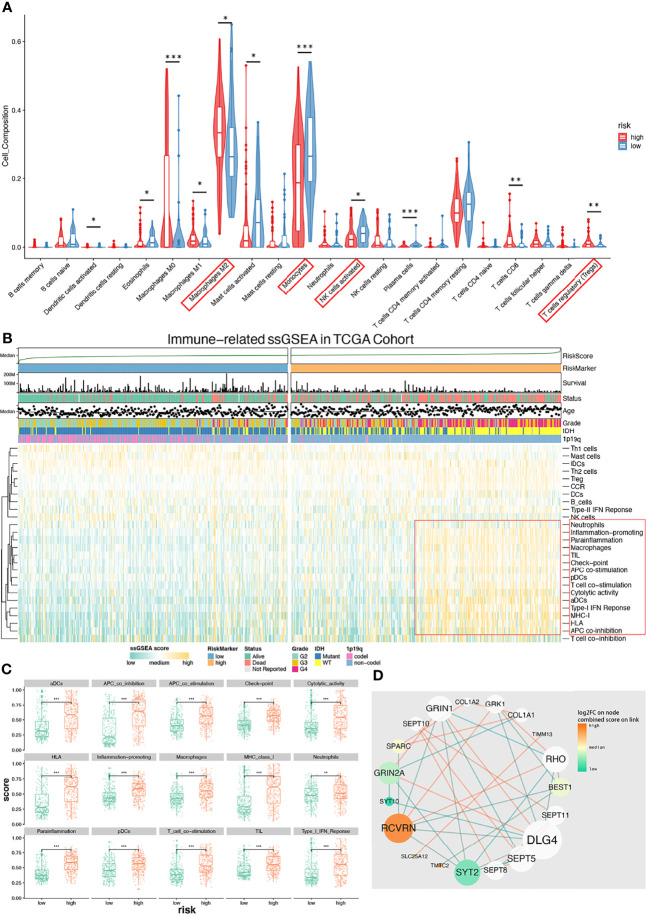
Immune microenvironment with low-/high-risk groups and PPI analysis network of CHGs. **(A)** Diagram of CIBERSORT result with low-/high-risk group. Immunosuppressive immunocyte (M2, Tregs) is significantly infiltrating in the high-risk group (p < 0.05), and activated NK cells is significantly infiltrating in the low-risk group (p < 0.05). **(B)** Immune-related ssGSEA heatmap shows that the immunosuppressive gene set (red box) was enriched in the high-risk group. **(C)** ssGSEA-enriched terms with low-/high-risk groups. **(D)** The PPI analysis of CHBs. This diagram shows that those immune-related terms are significantly (p < 0.01) enriched in the high-risk group. *p < 0.05, **p < 0.01, ***p < 0.001.

We next studied differences in the infiltrating immune cells between low-/high-risk groups. Results from CIBERSORT showed more active immunocytes in the high-risk group. Of note, myeloid cells with characteristics of immunosuppression showed the highest cell composition in samples (**Figure 6A**). Interestingly, there were more antitumor immunosuppressive cells in the high-risk group but less activated NK-activated cells (**Figure 6A**), possibly secondary to tumor-induced immunocyte exhaustion. Data (**Figure 6B**) from immune-related single-sample GSEA (ssGSEA) show that gene sets (red box) of myeloid-derived cells, including APC, DC, macrophages, and neutrophils, were significantly enriched. Those cells mainly have roles in antitumor pathways. Similarly, gene sets of checkpoint molecules and TIL (tumor-infiltrating lymphocytes) are also enriched in high-risk groups, which indicate effector T-cell deactivation under a higher expression of immune checkpoints like PD-1, TIM-3, and CTLA-4. We next compared enrichment scores of 15 gene sets (**Figure 6C**), with all of them receiving higher enriched scores in the high-risk group with p value < 0.001. The PPI analysis of CHGs was performed and shown in **Figure 6D**. We finally validated the expression levels of immune-suppressive markers (TGF-β/PD-L1) and found that both of the expression levels were increased as the tumor grade increased by IHC (**Supplementary Figure 5**). These results suggested that higher levels of the immune-suppressive microenvironment were correlated with higher grade and high-risk signature group.

## Discussion

Gliomas, particularly GBM, have been largely recalcitrant to current methods of treatment (18). According to the WHO criterion, glioma can be classified by the codeletion statue of 1p/19q, the mutation status of IDH, and the promoter of TERT. These molecular alterations always occur early during glioma formation and are regarded as a strong association with glioma patients’ overall survival (19). Patients with 1p/19q codeletion are sensitive to chemotherapy (20, 21). The mutation in IDH is associated with glioma metabolism (22), and the wild type of IDH is always associated with WHO IV (GBM) which is the most aggressive brain tumor and the poorest prognosis. The mutation in the promoter of TERT, which encodes telomerase, is also associated with GBM (23, 24). While calcium plays a critical role in both numerous physiological and pathological processes, previous studies examining the role of calcium in gliomas have been limited to mainly studying tumor invasion and migration (4, 25, 26).

The activity of calcium ions is getting more attention in glioma, owing to their key roles in many aspects in the pathophysiological process in glioma. For instance, studies indicated that calcium-dependent Cl (-) channels facilitate glioma cell invasion by promoting hydrodynamic cell shape and volume changes (27). Caren’s work showed that immune cells could regulate glioma invasion and migration *via* CCL5, which was influenced by the levels of intracellular and extracellular calcium ions (28). Besides, the summarized results indicate that glutamatergic and calcium signaling may provide positive feedback to promote glioma formation through (1) metabolic reprogramming and genetic switching to accelerate glioma duplication and progression and (2) upregulation of cytoskeleton proteins and elevation of intracellular Ca2+ levels to increase glutamate release and facilitate formation of synaptic-like connections with surrounding cells in their microenvironment (5). A novel mate-analysis from Robil (29) showed that store-operated calcium entry mechanisms in GBM and GBM stem cells appear different with normal brain tissue, and mitochondria may play a key role of calcium uptake mechanism in GBM stem cells.

In our study, we used the training cohort of TCGA-DEGs to select calcium-related BP and MF terms. Those terms included ion active/passivity transport, calcium-binding protein, signaling, cellular homeostasis, autophagy, apoptosis, and synaptic junctions. Comprehensively applying unsupervised/supervised learning yielded a risk score, an independent quantitative prognostic indicator which was independently verified by 2 external cohorts. Furthermore, a nomogram was established based on the risk score that was also validated. CIBERSORT and ssGSEA were used for examining infiltrating immune cells and immune-related gene sets. The findings in this study offer potential new biomarkers for predicting prognosis and evaluating the efficacy of immunotherapy in gliomas.

We have integrated the advantages of GO, KEGG, and GSEA, which not only focused on differentially expressed genes but also focused on significantly enriched gene sets with biological effects. Based on that, we obtained 460 calcium genes. CKGs were then found by Uni-Cox regression (**Supplementary Table 1**). Based on the result of consensus clustering, the best k usually depends on multiple factors; for our purposes, it was difficult to determine the best *k* according to the results among the CDF curve (**Figure 3B-I**), delta area (**Figure 3B-II**), and consensus matrix (**Figure 3B III-VIII**). Therefore, we performed a *k* prediction algorithm (**Figure 3C**) and found that *k* = 2 would be the best clustering number. Afterward, we conducted PCA and tSNE for a consensus stability test. Similar results were found from these two tests, and Cluster 1/2 was generated from unsupervised learning and was found to include different prognostic risk signatures (30) including grade, OS, SS, IDH status, 1p19q co-deletion status, and MGMT methylation status (**Figures 3C, E**). Those findings showed an advantage to unsupervised learning and identified patients with a good prognosis (Cluster2) and poor prognosis (Cluster1) and also indicated that CKGs play an important role in glioma progressing. We initially studied expression patterns of intersection (GL/GN/LN) TCGA-DEGs also within CKGs. CACNG3 and PRKCG were found to be highly expressed in Cluster 1. Meanwhile, SYT1, RYR2, and TRPC5 were expressed highly in Cluster 2. CACNG3 (calcium voltage-gated channel auxiliary subunit gamma 3) was reported as a predicted oncogene significantly dysregulated between GBM and normal control tissue (31, 32). PRKCG (protein kinase C) can be activated by calcium and second messenger diacylglycerol, which promotes cell migration in cancer (33). SYT1 (synaptotagmin 1), RYR2 (ryanodine receptor 2), TRPC5 (transient receptor potential cation channel subfamily C) regulated calcium transmembrane transport, stabilized cellular calcium homeostasis, and establishment of synaptic termini and upregulated autophagy (34, 35), which are suggested to participate in benign processes.

We created a risk score that has held up well as an independent prognostic indicator, which was established by supervised learning with calcium hub genes in the TCGA cohort. We also built nomograms to predict glioma patient survival. The calcium correlation network (**Figure 5H**) indicated SPARC, SLC25A12, GRIN2A, and SEPTIN5 as node genes (connection count greater than five). SPARC has already garnered interest as a multifaceted protein with a strong association with highly aggressive glioma (36). It impacts cancer growth in ambiguous ways in a context-dependent manner (37). SPARC has been used as a biomarker for both diagnosis and prognosis (38) and functions as a sensitizer to chemotherapy by enhancing apoptosis with interfering activity of Bcl-2 (39) in colon cancer. In glioma, SPARC can suppress tumor growth but promote invasion and migration by regulating integrin and growth factor receptor-regulated kinases with their downstream effectors (40). Data from our study also indicated that SPARC is expressed at much higher levels in mesenchymal GBM subtypes (**Supplementary Figure 3E**). Besides, in Leclerc and colleagues’ study (3), the concept of cell competition was involved and SPACR was defined as a marker of “loser cell” that obtained a lower rate of proliferation. Their research also indicated that SPARC increases invasion and survival while inhibiting proliferation. SEPTIN5 (also known as SEPT5) has been found to be involved in forming vesicle membranes and appear to be important for vesicle transport machinery; the septin complex affects the cytosolic Ca2^+^ by downregulating the expression of ORAI and IP3R (41, 42). SEPTIN7 is downregulated in gliomas, and its decreased expression negatively correlates with increased tumor grade (43). Overexpression of SEPTIN7 inhibits cell proliferation and arrests cell cycle in the G0/G1 phase both in *vitro* and in *vivo*. SEPTIN7 knockouts in glioma xenografts result in accelerating tumor growth (44). Moreover, previous research shows that overexpression of SEPTIN 7 suppresses glioma growth (44). Similarly, our study found that the expression value of SEPTIN5 drastically decreases as the grade increases (**Supplementary Figure 3A**) and patients with a higher expression of SEPTIN5 are associated with longer survival (**Supplementary Figures 3H–V**). GRIN2A (glutamate ionotropic receptor NMDA type subunit 2A) encodes a protein that belongs to the glutamate-gated ion channel protein family, whose activation results in a calcium influx. The protein of GRIN2A is a calcium sensor that participates in triggering neurotransmitter release at the synapse. Several studies show how mutants of GRIN2A result in malignant melanoma (44, 45). Glutamate receptors have been linked with tumorigenesis in glioma (46). Our data show that a higher expression of GRIN2A is associated with better prognosis (**Supplementary Figures 3A, E, H–III**). SLC25A12 (calcium-binding mitochondrial carrier protein Aralar1) plays roles in transporting cytoplasmic glutamate with mitochondrial aspartate across the inner mitochondrial membrane as an antiporter upon binding of a calcium ion. Lack of SLC25A12, an important component of the malate-aspartate carrier, impairs cytosolic aspartate levels, NAD+/NADH ratio, mitochondrial respiration, and tumor growth (47), which is considered to be related with metabolism. It has a higher expression with IDH mutant (**Supplementary Figure 3D**), lower grade (**Supplementary Figure 3A**), and longer survival (**Supplementary Figure 3H–VII**).

PPI analysis indicates that DLG4 has the highest degree value followed by RCVRN, GRIN2A, and BEST1 in this network (**Figure 6D**). However, DLG4 is a predicted gene generated by PPI analysis. RCVRN encodes recoverin (also known as cancer-associated retinopathy protein), which is a retina-specific calcium ion-binding protein normally only expressed in neurons in the eye (48). Our data show that a high expression of RCVRN is correlated with poor prognosis risk signatures as well as shorter survival (**Supplementary Figures 3B–F, H–IV**). Cancer-associated retinopathy is usually caused by recoverin, in which aberrant expression can activate a host immune response followed by development of a paraneoplastic neurological syndrome (49, 50). Recoverin levels were detected 10-fold higher in recurrent GBM patients relative to controls (51) and were also considered as a potential circulating glioma biomarker (52). Unfortunately, there is no clinical information about glioma-related oculopathy among the 3 cohorts we used; as such, it remains unknown whether these data correlate with any paraneoplastic syndromes. BEST1 encodes bestrophin-1, which functions as a calcium-activated chloride channels regulator of intracellular Ca2^+^, and the expression is highest in the retina in humans. Bestrophin-1 may contribute to volume regulation in particular cell types, including glioma cells (7, 53).

Calcium-activated chloride channels can play a vital role in cell volume regulation, resulting in migration. Studies show that bestrophin-1 is implicated in tumor suppression by a proapoptotic mechanism in breast cancer (54). There are studies that suggest that the function of bestrophin-1 has an ion dependent context (55). In our study, the expression value of BEST1 is higher in better prognostic risk signature groups (**Supplementary Figures 3A–D, F**), such as IDH wild type, low-risk group, and Cluster 2. However, we observed a higher expression in the GBM subtype of mesenchymal than in classical (**Supplementary Figure 3E**). To our knowledge, mesenchymal is a subtype of high invasion and migration, and one of the reasons may be from bestrophin-1’s function in regulating cell volume.

Based on the potential function we observed from the aberrant expression of RCVRN in immune response, we observed that more myeloid-derived cells, Tregs (T regular cells), and checkpoint molecules were enriched in the high-risk group. Previous studies have demonstrated a poor OS with immunosuppressive phenotypes in glioma (56–58) due to their implications in dysfunctional CD8^+^ T cell and angiogenesis. Interestingly, APCs (antigen-presenting cells) and DC (dendritic cell) gene sets were enriched in the high-risk group. As the recoverin is aberrantly generated and a potential cancer retina antigen (59, 60) is released into the glioma microenvironment, it is likely that the tumor microenvironment is attracting APCs and DCs. Moreover, the checkpoint gene set is correlated with the high-risk group, representing glioma evading immune surveillance and poor clinical outcomes. However, despite these immune barriers, the possibility of checkpoint blockade remains a viable option (61).

There are several limitations in our study. Our prognosis-predicted model was constructed by the TCGA training cohort and validated by 2 independent external validation cohorts, CGGA and Rembrandt. Further clinical glioma patient data are warranted to promote clinical usability. Some clinical features of the samples we used were missing, which shrank the total sample size to maintain the fidelity of the cohorts. Finally, validation of our results beyond a clinical dataset would lend further credence to the utility of our proposed gene sets in targeting glioma.

In conclusion, we present a comprehensive analysis with an unsupervised and supervised learning approach to predict glioma patient prognosis as related to calcium-related genes. Our approach generated an easy-to-use nomogram for clinicians to evaluate glioma patients’ prognosis with the potential to inform treatments for this difficult-to-treat disease.

## Data Availability Statement

The original contributions presented in the study are included in the article/**Supplementary Material**. Further inquiries can be directed to the corresponding authors.

## Ethics Statement

The studies involving human participants were reviewed and approved by the Ethical Committee of Tianjin Medical University General Hospital. The patients/participants provided their written informed consent to participate in this study. Written informed consent was obtained from the individual(s) for the publication of any potentially identifiable images or data included in this article.

## Author Contributions

Conceptualization, PL, YL, YZ, ML, and XY. Data curation, YZ, JZ, GS, and LZ. Formal analysis, PL and GS. Investigation, PL, YL, YZ, JC, JZ, and GS. Methodology, BL and LS. Resources, SY. Software, PL and BL. Supervision, ML and XY. Validation, Y-JL. Visualization, YL, Y-JL, and LS. Writing—original draft, PL and YL. Writing—review and editing, JC, LY, ML, and XY. All authors contributed to the article and approved the submitted version.

## Funding

This work was supported by funding from the National Natural Science Foundation of China (No. 81872063), Beijing-Tianjin-Hebei Basic Research Cooperation Project (No. 19JCZDJC64200), and Scholarship funding from the China Scholarship Council (No. 201906940040).

## Conflict of Interest

The authors declare that the research was conducted in the absence of any commercial or financial relationships that could be construed as a potential conflict of interest.

## Publisher’s Note

All claims expressed in this article are solely those of the authors and do not necessarily represent those of their affiliated organizations, or those of the publisher, the editors and the reviewers. Any product that may be evaluated in this article, or claim that may be made by its manufacturer, is not guaranteed or endorsed by the publisher.

## Supplementary Material

Supplementary Figure 1 Differentially expressed genes (DEGs) and enrichment analysis in GBM-Normal, GBM-LGG and LGG-Normal **(A, B):** DEGs in GBM-LGG **(A, I.)**, GBM-Normal **(A-II.)** and LGG-Normal groups **(A-III.)** and the intersection. **(C, D)**: KEGG analysis calcium pathway (hsa04010) in GBM-LGG **(C-I.)**, GBM-Normal **(C-II.)**, LGG-Normal **(C-III.)**. **(E)**: Enriched heatmap of calcium-related biological processes. **(F)**: GSEA running score of calcium-related molecular function. **(I.)** GBM-Normal, **(II.)** LGG-Normal.

Supplementary Figure 2 Results of consensus clustering **(A, B)**: Consensus clustering matrix for k = 8 **(A)**, k = 9 **(B)**. **(C)**: The tracking plot for k = 2~9. **(D, E)**: The histogram for k = 2 **(D)**, k = 3 **(E)**. **(F)**: Weight bar chart for k = 2~9.

Supplementary Figure 3 Calcium Hub genes expression levels with clinical feature, high/low-risk groups and Cluster ½ **(A–F)**: Expression levels of Calcium Hub genes with Grades **(A)**, Cluster 1/2 **(B)**, high/low-risk groups **(C)**, IDH status **(D)**, GBM subtypes **(E)**, LGG IDH status **(F)**. **(G)**: Kaplan-Meier survival curve of Calcium Hub Genes **(I–X.)**.

Supplementary Figure 4 Validation for risk score with external independent (CGGA-324 and Rembrandt) cohort **(A, F)**: Kaplan–Meier overall survival curves for high/low-risk groups in CGGA-325 **(A)** and Rembrandt **(F)** cohort. **(B, G)**: ROC curve showed a satisfied predictive efficiency of the 1/3/5-year survival rate on risk score both in CGGA-325 **(B)** and Rembrandt **(G)** cohort. **(D, E)**: Multi-Cox and logistic regression Nomogram of CGGA-325 cohort with independent predictor. **(C)**: Calibrate curves of Mulit-Cox regression Nomogram **(I-III.)** and logistic regression Nomogram **(IV.)** in CGGA-325 cohort show a satisfied predicted efficiency. **(I, J)**: Multi-Cox and logistic regression Nomogram of Rembrandt cohort with independent predictor. **(H)**: Calibrate curves of Mulit-Cox regression Nomogram **(I-III.)** and logistic regression Nomogram **(IV.)** in Rembrandt cohort also show a satisfied predicted efficiency.

Supplementary Figure 5 Immunohistochemistry (IHC) of PD-L1 and TGF-β in diffuse glioma (grade II-IV) and control tissue.
