# Supplementary figures and images for: Calcium-Related Gene Signatures May Predict Prognosis and Level of Immunosuppression in Gliomas
Source: Front Oncol. 2022 May 13;12:708272. doi: 10.3389/fonc.2022.708272 (PMC9136236; doi:10.3389/fonc.2022.708272)

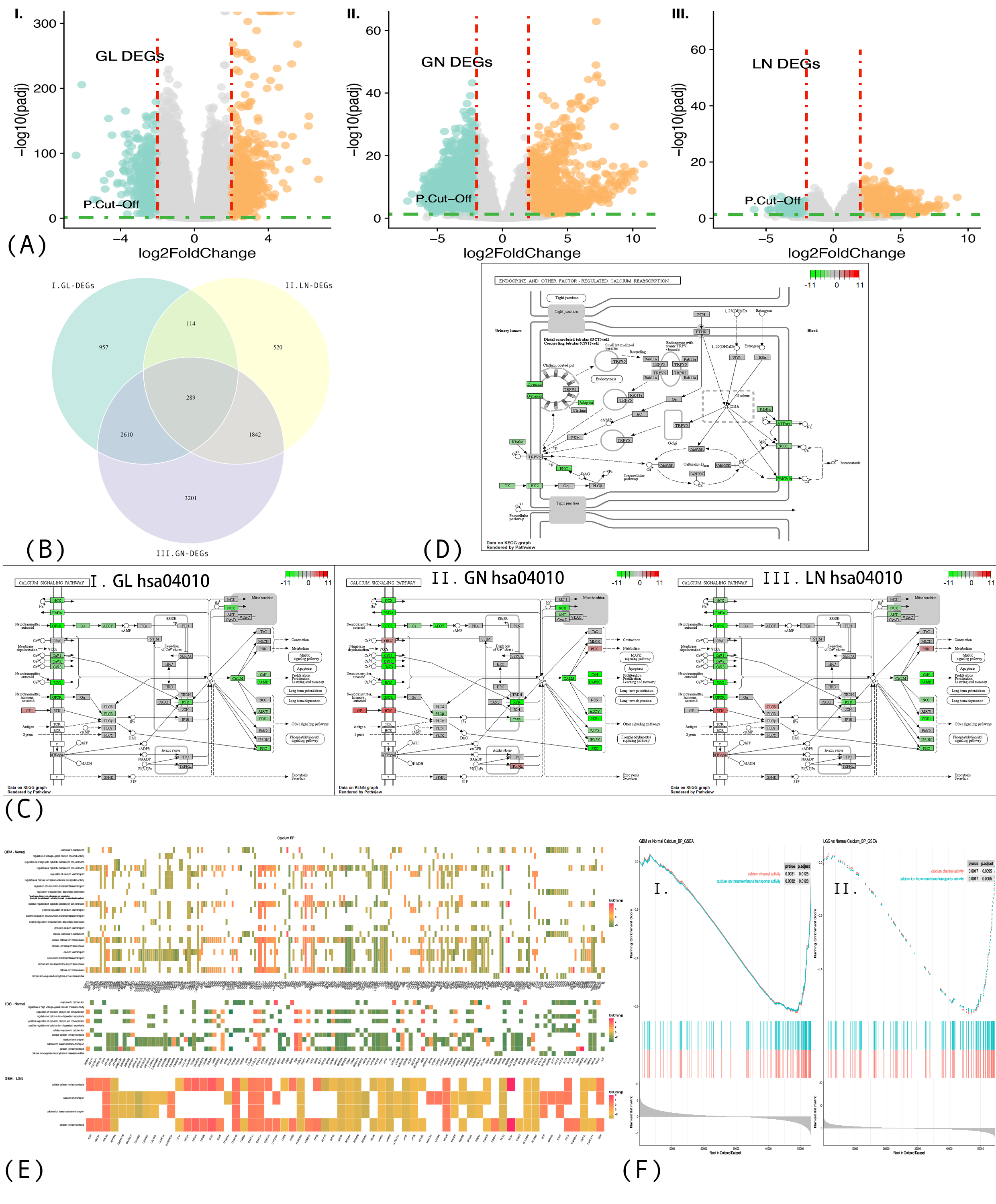

Supplement: Supplementary Figure 1 — Differentially expressed genes (DEGs) and enrichment analysis in GBM-Normal, GBM-LGG and LGG-Normal (A, B): DEGs in GBM-LGG (A, I.), GBM-Normal (A-II.) and LGG-Normal groups (A-III.) and the intersection. (C, D): KEGG analysis calcium pathway (hsa04010) in GBM-LGG (C-I.), GBM-Normal (C-II.), LGG-Normal (C-III.). (E): Enriched heatmap of calcium-related biological processes. (F): GSEA running score of calcium-related molecular function. (I.) GBM-Normal, (II.) LGG-Normal. [file Image_1.tif]

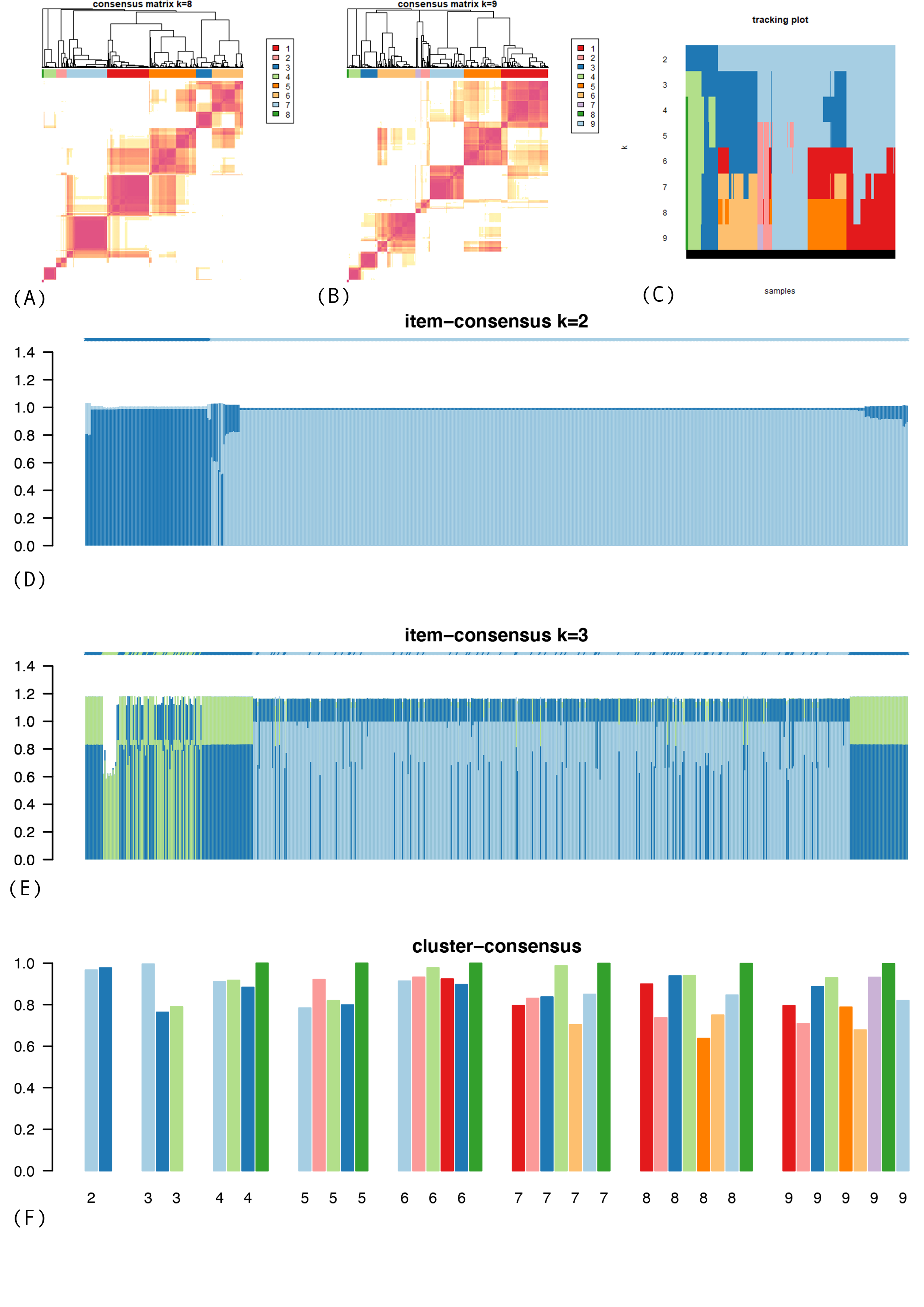

Supplement: Supplementary Figure 2 — Results of consensus clustering (A, B): Consensus clustering matrix for k = 8 (A), k = 9 (B). (C): The tracking plot for k = 2~9. (D, E): The histogram for k = 2 (D), k = 3 (E). (F): Weight bar chart for k = 2~9. [file Image_2.tif]

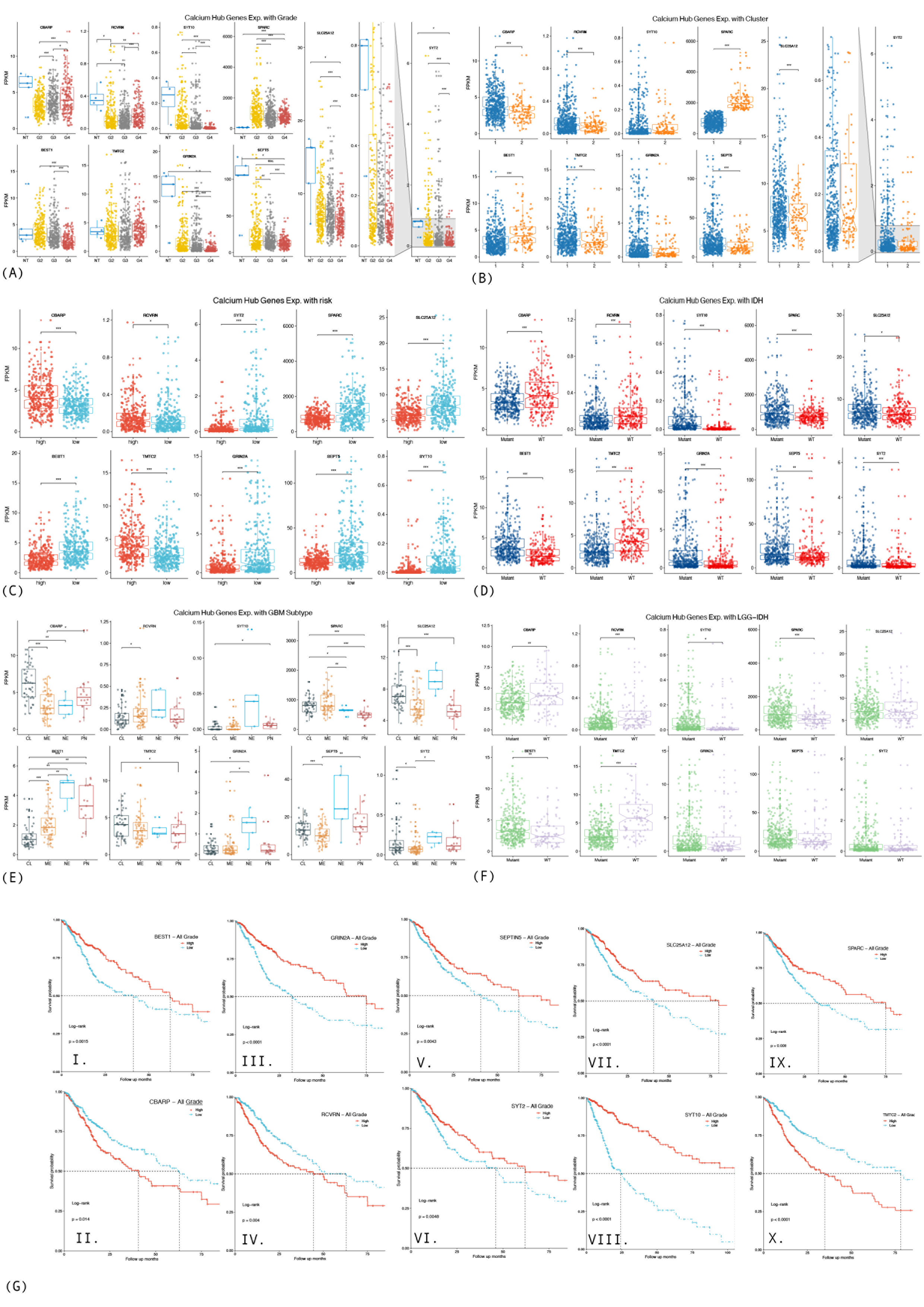

Supplement: Supplementary Figure 3 — Calcium Hub genes expression levels with clinical feature, high/low-risk groups and Cluster ½ (A–F): Expression levels of Calcium Hub genes with Grades (A), Cluster 1/2 (B), high/low-risk groups (C), IDH status (D), GBM subtypes (E), LGG IDH status (F). (G): Kaplan-Meier survival curve of Calcium Hub Genes (I–X.). [file Image_3.tif]

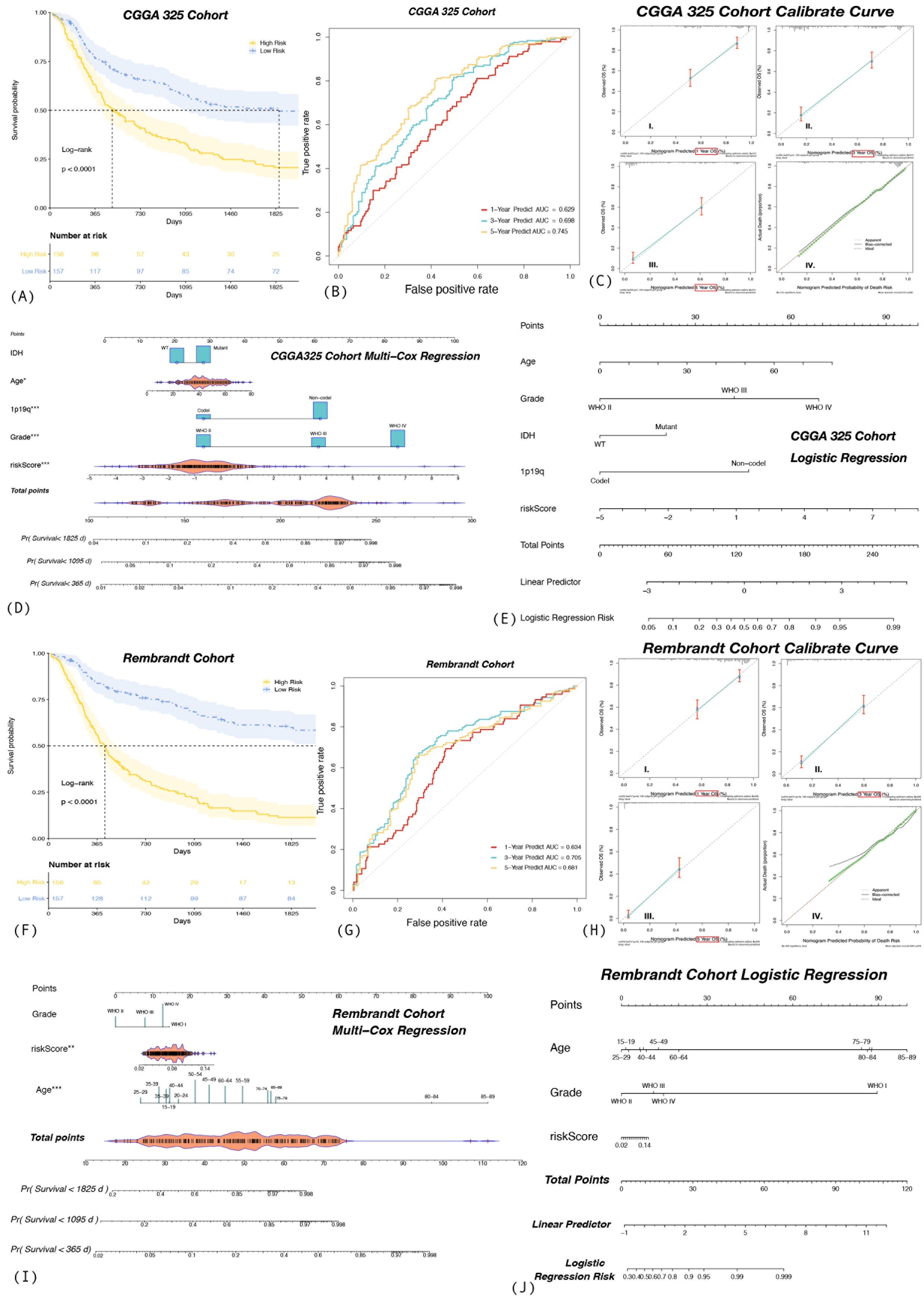

Supplement: Supplementary Figure 4 — Validation for risk score with external independent (CGGA-324 and Rembrandt) cohort (A, F): Kaplan–Meier overall survival curves for high/low-risk groups in CGGA-325 (A) and Rembrandt (F) cohort. (B, G): ROC curve showed a satisfied predictive efficiency of the 1/3/5-year survival rate on risk score both in CGGA-325 (B) and Rembrandt (G) cohort. (D, E): Multi-Cox and logistic regression Nomogram of CGGA-325 cohort with independent predictor. (C): Calibrate curves of Mulit-Cox regression Nomogram (I-III.) and logistic regression Nomogram (IV.) in CGGA-325 cohort show a satisfied predicted efficiency. (I, J): Multi-Cox and logistic regression Nomogram of Rembrandt cohort with independent predictor. (H): Calibrate curves of Mulit-Cox regression Nomogram (I-III.) and logistic regression Nomogram (IV.) in Rembrandt cohort also show a satisfied predicted efficiency. [file Image_4.tif]

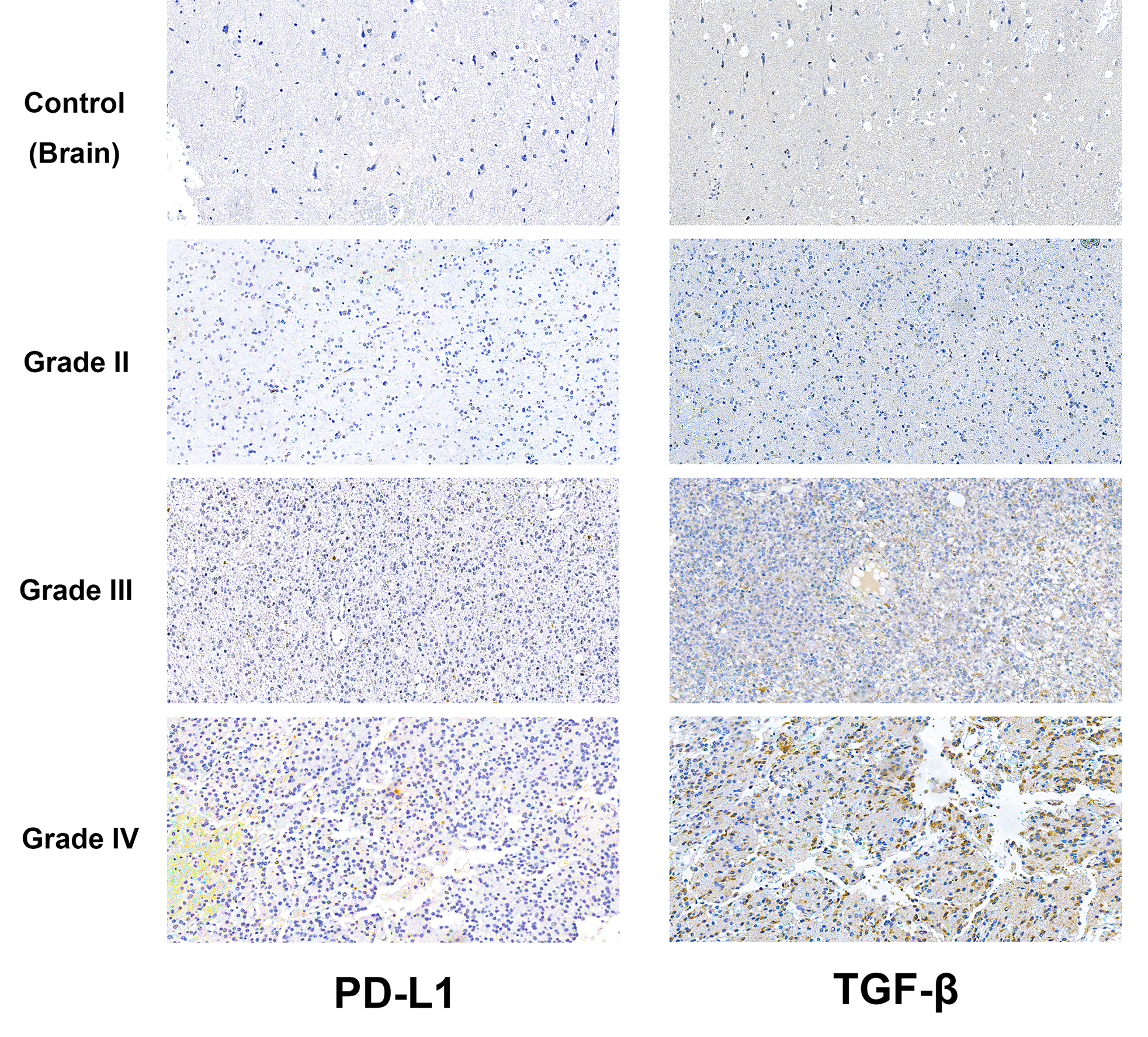

Supplement: Supplementary Figure 5 — Immunohistochemistry (IHC) of PD-L1 and TGF-β in diffuse glioma (grade II-IV) and control tissue. [file Image_5.tif]
