# Supplementary material for: Calcium-Related Gene Signatures May Predict Prognosis and Level of Immunosuppression in Gliomas
Source: Front Oncol. 2022 May 13;12:708272. doi: 10.3389/fonc.2022.708272 (PMC9136236; doi:10.3389/fonc.2022.708272)
Supplement: Supplementary file 6 [file Table_1.docx]

| **id** | **coef** | **HR** | **HR.95L** | **HR.95H** | **pvalue** |
| --- | --- | --- | --- | --- | --- |
| TPH2 | -11.946 | 6.49E-06 | 1.40E-08 | 0.003005 | 0.000137 |
| HCRTR2 | -6.46652 | 0.001555 | 4.29E-05 | 0.056374 | 0.000416 |
| SYT10 | -4.89613 | 0.007475 | 0.000572 | 0.097729 | 0.000189 |
| RASA4B | -4.16477 | 0.015533 | 0.001656 | 0.145694 | 0.000266 |
| HCRTR1 | -3.09299 | 0.045366 | 0.00838 | 0.245583 | 0.000331 |
| TRPC5 | -2.24119 | 0.106332 | 0.032721 | 0.345541 | 0.000194 |
| PTGER3 | -2.1754 | 0.113563 | 0.036052 | 0.357721 | 0.000202 |
| LCE1D | -2.076 | 0.125431 | 0.042759 | 0.36794 | 0.000156 |
| KCNMB2 | -0.63785 | 0.528425 | 0.37533 | 0.743967 | 0.000258 |
| SYT2 | -0.52642 | 0.590716 | 0.436869 | 0.798743 | 0.000627 |
| PRKAA2 | -0.5194 | 0.594875 | 0.437772 | 0.808357 | 0.000901 |
| RYR2 | -0.4066 | 0.665909 | 0.533482 | 0.831209 | 0.000325 |
| PCLO | -0.36634 | 0.693266 | 0.56248 | 0.854463 | 0.000594 |
| CPNE9 | -0.33478 | 0.715498 | 0.586416 | 0.872993 | 0.000973 |
| FGF14 | -0.26259 | 0.769057 | 0.661897 | 0.893566 | 0.000604 |
| CACNB2 | -0.22774 | 0.796333 | 0.707689 | 0.896082 | 0.000155 |
| GRIN2A | -0.14534 | 0.864731 | 0.793531 | 0.94232 | 0.000916 |
| BEST1 | -0.14382 | 0.866041 | 0.797506 | 0.940466 | 0.000628 |
| SLC25A12 | -0.11242 | 0.893668 | 0.839692 | 0.951113 | 0.000405 |
| DNM1L | -0.08664 | 0.917005 | 0.877324 | 0.958482 | 0.000124 |
| CACNG3 | -0.08261 | 0.920707 | 0.878142 | 0.965334 | 0.000624 |
| PRKCG | -0.0602 | 0.941574 | 0.908919 | 0.975402 | 0.000829 |
| RIMS3 | -0.04592 | 0.955121 | 0.930383 | 0.980517 | 0.000605 |
| SNCA | -0.03324 | 0.967303 | 0.948547 | 0.98643 | 0.000876 |
| SEPTIN5 | -0.0259 | 0.974431 | 0.961403 | 0.987635 | 0.000162 |
| GOT1 | -0.025 | 0.975308 | 0.962382 | 0.988407 | 0.00024 |
| NAPB | -0.02142 | 0.978805 | 0.967793 | 0.989942 | 0.000206 |
| RAB3A | -0.01499 | 0.985126 | 0.9776 | 0.99271 | 0.000128 |
| SYT1 | -0.01216 | 0.98791 | 0.981203 | 0.994663 | 0.000466 |
| PRNP | -0.00682 | 0.993205 | 0.989499 | 0.996926 | 0.000352 |
| SNAP25 | -0.00397 | 0.996036 | 0.993964 | 0.998111 | 0.000184 |
| SPARC | -0.00049 | 0.99951 | 0.999243 | 0.999778 | 0.000329 |
| TMTC2 | 0.07721 | 1.080269 | 1.038744 | 1.123454 | 0.000113 |
| CBARP | 0.118256 | 1.125532 | 1.055426 | 1.200294 | 0.000313 |
| RCVRN | 0.856804 | 2.355621 | 1.446331 | 3.83657 | 0.000576 |

**Supplementary Table 1: Uni-Cox result of Calcium Key Genes (*P* < 0.001)**
