# Supplementary material for: Calcium-Related Gene Signatures May Predict Prognosis and Level of Immunosuppression in Gliomas
Source: Front Oncol. 2022 May 13;12:708272. doi: 10.3389/fonc.2022.708272 (PMC9136236; doi:10.3389/fonc.2022.708272)
Supplement: Supplementary file 7 [file Table_2.docx]

| ID |
| --- |
| GO:0055074 |
| GO:0006816 |
| GO:0006874 |
| GO:0070588 |
| GO:0017156 |
| GO:0017158 |
| GO:0051480 |
| GO:0007204 |
| GO:0051592 |
| GO:0045956 |
| GO:0051924 |
| GO:0051928 |
| GO:0071277 |
| GO:1901385 |
| GO:0051482 |
| GO:0048791 |
| GO:0060402 |
| GO:1903169 |
| GO:0099509 |
| GO:0060401 |
| GO:0097553 |
| GO:1904427 |
| GO:1901019 |
| GO:1901841 |
| GO:0051209 |
| GO:0051283 |
| GO:0051282 |
| GO:0051208 |
| GO:0019722 |

Table 2. Calcium-related gene sets
